# Size-Dependent Gold Nanoparticle Interaction at Nano–Micro Interface Using Both Monolayer and Multilayer (Tissue-Like) Cell Models

**DOI:** 10.1007/s40820-015-0060-6

**Published:** 2015-09-15

**Authors:** Darren Yohan, Charmainne Cruje, Xiaofeng Lu, Devika B. Chithrani

**Affiliations:** 1 Department of Physics, Ryerson University, 350 Victoria Street, Toronto, ON M5B 2K3 Canada; 2 Keenan Research Centre, Li Ka Shing Knowledge Institute, St. Michael’s Hospital, Toronto, ON Canada

**Keywords:** Gold nanoparticles, Multilayer cellular structures, Tissue penetration, Hyper spectral imaging, Size dependence

## Abstract

Gold nanoparticles (GNPs) are emerging as a novel tool to improve existing cancer therapeutics. GNPs are being used as radiation dose enhancers in radiation therapy as well as anticancer drugs carriers in chemotherapy. However, the success of GNP-based therapeutics depends on their ability to penetrate tumor tissue. GNPs of 20 and 50 nm diameters were used to elucidate the effects of size on the GNP interaction with tumor cells at monolayer and multilayer level. At monolayer cell level, smaller NPs had a lower uptake compared to larger NPs at monolayer cell level. However, the order was reversed at tissue-like multilayer level. The smaller NPs penetrated better compared to larger NPs in tissue-like materials. Based on our study using tissue-like materials, we can predict that the smaller NPs are better for future therapeutics due to their greater penetration in tumor tissue once leaving the leaky blood vessels. In this study, tissue-like multilayer cellular structures (MLCs) were grown to model the post-vascular tumor environment. The MLCs exhibited a much more extensive extracellular matrix than monolayer cell cultures. The MLC model can be used to optimize the nano–micro interface at tissue level before moving into animal models. This would accelerate the use of NPs in future cancer therapeutics.

**Electronic supplementary material:**

The online version of this article (doi:10.1007/s40820-015-0060-6) contains supplementary material, which is available to authorized users.

## Introduction

**Fig. 1:**
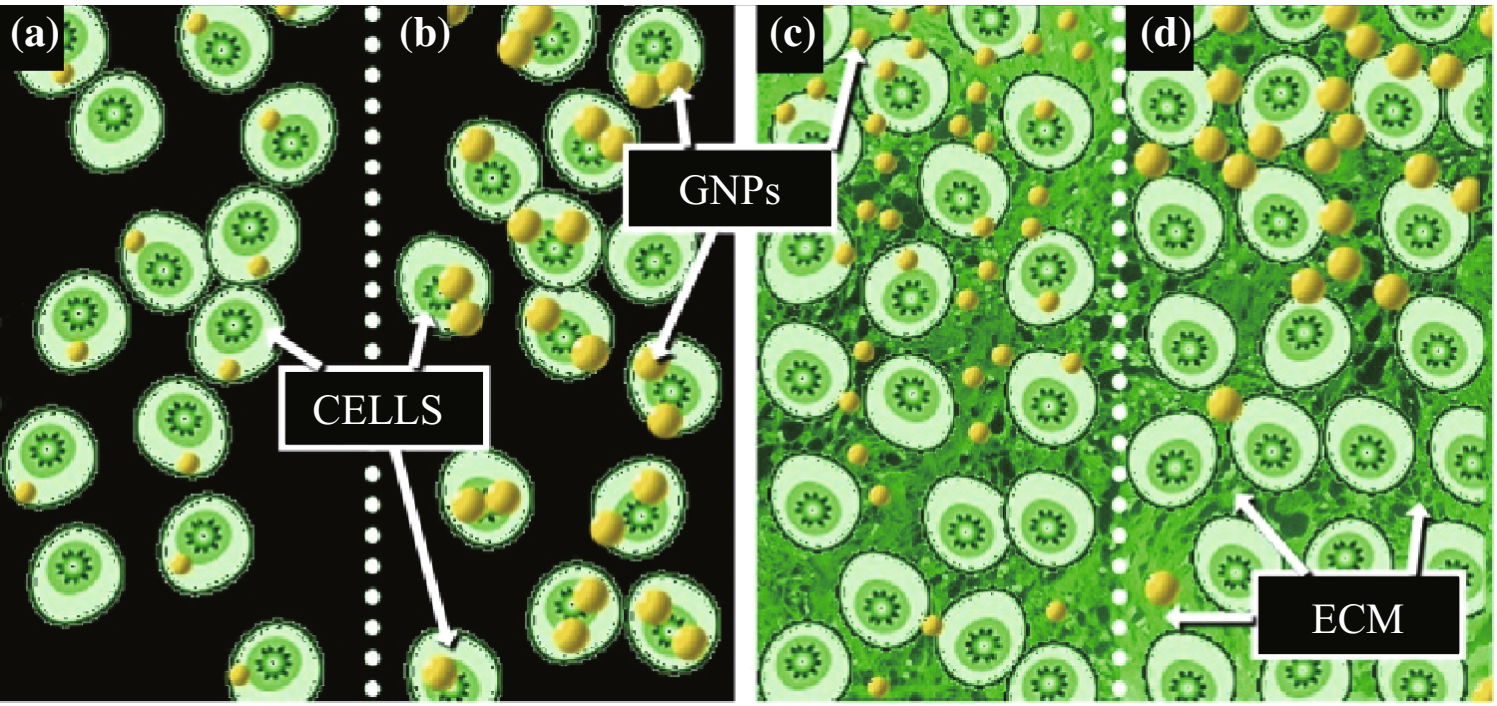
Schematic explaining the size dependency of NP transport in monolayer versus multilayer. **a** Cellular uptake of smaller NPs is lower at monolayer level. **b** Larger GNPs have greater cell uptake than smaller GNPs at the monolayer level. **c** Smaller GNPs have better penetration in the multilayer tissue than larger GNPs. **d** Larger GNPs exhibit limited penetration through ECM

Gold nanoparticles (GNPs) and other NPs have emerged as one of the most robust and flexible tools in cancer research. Their applications cover the full breadth of the clinical spectrum from enhancing diagnostic imaging to photothermal and photodynamic therapy, drug delivery, and radiosensitization [1, 2]. NPs were found to enter cells mostly via receptor-mediated endocytosis (RME) [3]. At the monolayer level, GNP uptake is size dependent [4]. For example, smaller NPs have a lower uptake compared to larger NPs as illustrated in Fig. 1. However, most of the outcomes related to NP uptake and transport derived from monolayer cell cultures ultimately fall short of agreeing with the outcomes derived from an in vivo tumor environment. Hence, the success of several NP-based therapies and systems is sometimes overstated and in vivo trials fall short of expectations [5, 6]. In this study, a new multilayer cell culture is used in an attempt to bridge the gap between the in vitro and in vivo studies.

In post-vascular tumor tissue, the local environment is much more complex than in monolayer cell cultures due to the presence of the extracellular matrix (ECM). The ECM is a structural and biological support network that arises from proteins and molecules secreted by cells as they grow. The composition and architecture of the ECM vary from one cell line to another since different cells secrete different proteins with different functions. The ECM carries out a number of important functions, enables cell-to-cell communication and cell adhesion, and provides a rigid support structure that gives tissues its shape. This support structure also determines the permeability of tissue to molecules and other particles and sometimes acts as a barrier for transport, as discussed in the next section [7–10]. Eosin is a florescent dye used for staining proteins in the cytoplasm of the cells and collagen in the ECM. As illustrated in Fig. 1, ECM (marked in green) is not heavily present in between cells compared to tissues. Hence, ECM does not act as a barrier for NP transport in monolayer cell cultures whereas it does for NP transport in tissue-like MLC structures. For example, it was shown that NP penetration into the core tumor spheroids diminished as particle sizes increased from 20 to 100 nm NPs [9]. Several factors have been proposed to explain the poor penetration and distribution of GNPs in solid tumors. These factors include the binding of GNPs to tumor tissue, increased interstitial fluid pressure, and hindered diffusion due to cell packing and the ECM [7, 8, 11–13]. Furthermore, the elimination of the pressure gradient in solid tumors yielded only minimal gains in macromolecule delivery which suggests that cell packing and ECM are major factors in the differences between monolayer and solid tumor penetration dynamics [8, 12, 14].

The geometry of the MLCs also sets it apart from other 3D cell cultures, such as the multicellular spheroid, as discussed in the previous section. In the spheroid geometry, drugs and agents pass radially inwards from the edges of the spheroid. This geometry is often the reverse of in vivo physiology and makes it difficult to ascertain the penetration of drugs in a solid tumor. The MLC provides a top-down or side-to-side geometry and better mimics the distribution of agents out of a blood vessel and down into tissue [15–17]. In addition, it has been reported that within tumors, cells can appear further than 150 µm from the nearest blood vessel [18]. In such a case, any agents delivered to the tumor through the bloodstream will have to navigate in the heterogeneous tumor environment without assistance from any vascular network to reach their target cells. Our proposed MLC model can also be used to examine the penetration and transport of GNPs through such tissue.

While in vivo tumors offer the most clinically relevant tool for determining the spatial distribution and penetration of GNPs, the complex biological factors prevent a focused analysis of the individual components that contribute to the transport of GNPs and other agents [19–21]. Using this MLC model, the limiting effects of the ECM, the thickness of cell layers, and the GNP transport mechanisms of GNPs in tumor tissue can be measured in a less complex biological microenvironment. In this study, we investigated the size dependency of NP transport in tumor tissues using the MLC model for the first time. We were able to image the penetration of NPs in these tissue sections for the first time. The accumulation of GNPs in the MLC tissue was measured to determine the major factors affecting GNP transport through tumor tissue in the absence of vasculature. Hence, the MLC model can be used to improve the design of NP-based systems for improved outcomes in imaging and therapeutic applications in the field of nanomedicine.

## Materials and Methods

### Selection of Cell Lines

In this study, two cell lines, MDA-MB-231 and MCF-7, were chosen as a model tumor type. Both cell lines are invasive ductal carcinomas and thus represent one of the most commonly encountered types of cancers. This also means that both cell lines are derived from the same tissue which helps limit the potential variations between tissue types. In addition, both cell lines have comparable doubling times and sizes. However, the MDA-MB-231 cell line has been identified as being far more aggressive and invasive than the MCF-7 cell line [22–28]. This key difference affects the makeup and proliferation of not only the cells themselves but also the ECM that surrounds them.

### Synthesis of GNPs

The GNPs were synthesized via the reduction of HAuCl_4_ by sodium citrate, which is more commonly referred to as the Turkevitch’s method [29]. By varying the amount of sodium citrate, this method can yield NPs of varying sizes. In summary, 300 µL of 1 % gold salt was added to 30 mL of doubled distilled water and brought to boil while stirring. The amount of reducing agent (1 % sodium citrate) added was 400 and 100 µL to produce 20 and 50 nm, respectively. The GNPs were characterized by transmission electron microscopy (TEM, H7000, Hitachi Corp. Tokyo, Japan), UV-spectroscopy (Lambda 40; PerkinElmer, Waltham, MA), and dynamic light scattering (DLS) using 90 Plus Particle Sizer Analyzer (Brookhaven Instruments Corp. New York, NY) to determine the size of the particles. The UV–visible peak wavelengths for 20 and 50 nm GNPs were 520 and 525 nm, respectively. According to TEM, the diameters of the NPs were 19.5 (±3.3) and 52.4 (±5.2). According to DLS measurements, the hydrodynamic diameters of the NPs were 24.2 (±0.6) and (52.6 ± 0.8). This information is given under supplementary information as well.

### The CytoViva Imaging Technology

**Fig. 2:**
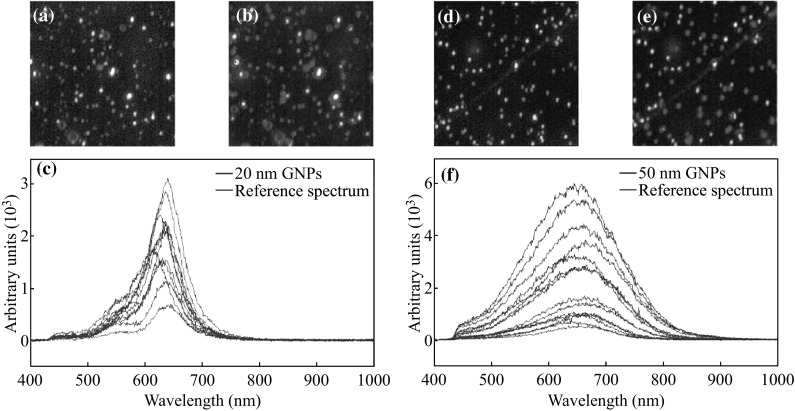
Characterization of GNPs. **a**, **b**, **c** The dark-field hyperspectral image, mapped image using the reference spectra marked in *red* in (**c**), and reflectance spectra of GNPs of size 20 nm, respectively. **d, e, f** The dark-field hyperspectral image, mapped image using the reference spectra marked in *red* in (**f**), and reflectance spectra of GNPs of size 50 nm, respectively. (Color figure online)

The CytoViva technology used in this study was specifically designed for optical observation and spectral confirmation of NPs as they interact with cells and tissues. The illumination of the microscope system utilizes oblique angle illumination to create high-resolution dark-field images. Figure 2a, d shows dark-field hyperspectral images of GNPs of sizes 20 and 50 nm. The GNPs appear bright owing to their high scattering cross section. Hyperspectral imaging (HSI) was used in conjunction with the dark-field microscope to obtain reflectance spectra from each pixel in the dark-field image. Spectral angle mapping can be performed to conduct a pixel-by-pixel matching of any spectra obtained by the system. This procedure was used to create a map of GNPs based on their reflectance spectra within the sample. The hyperspectral image shows which HSI pixels matched the reference GNP spectrum within a given spectral angle threshold set at 0.15 radians for this study. Figure 2b, e shows the hyperspectral images with an overlaid spectral angle map where the red dots represent matching GNP reference spectra. The reference spectrum was chosen from a sample of GNP spectra collected via the HSI image. It is representative of a typical GNP spectrum for the sample studied. Figure 2c, f shows the spectral information for a few GNPs in the Fig. 2a, d. The reference spectrum marked in red in Fig. 2c, f was used to map the GNP distribution in Fig. 2b, e. This HIS imaging of GNPs in cells and tissues has been very practical since it is not necessary to optically label NPs. Furthermore, it is also possible to extract spectral info from each pixel to verify whether the bright spots appears in the sample image are actually GNPs. This imaging technique requires less preparation in comparison to TEM and is also cost effective.

### Growth of MCL Structures

To better model the tumor environment, cells were cultured on a semi-permeable membrane, as described by Minchinton et al. [17]. The multi-layered cell culture develops into a thick mat of cells that acts as a barrier to the penetration of drugs and GNPs much in the same way as solid tumors and multicellular spheroids. They have been used to study the penetration of a variety of anticancer drugs as well as agents specifically targeted to hypoxic and nutrient deprived cells, where penetration is of the utmost importance [16]. Previous work using MLCs demonstrated the development of hypoxia and necrosis in MLCs greater than 150-µm thick. The appearance of necrosis and hypoxia at 150 µm of tissue thickness presents an upward bound on the tissue sizes considered in this study [15, 18, 30–33]. The effects of hypoxia and necrosis on the transport of GNPs were shown recently to be quite complex and will be pursued at the multilayer level in future studies [34].

**Fig. 3:**
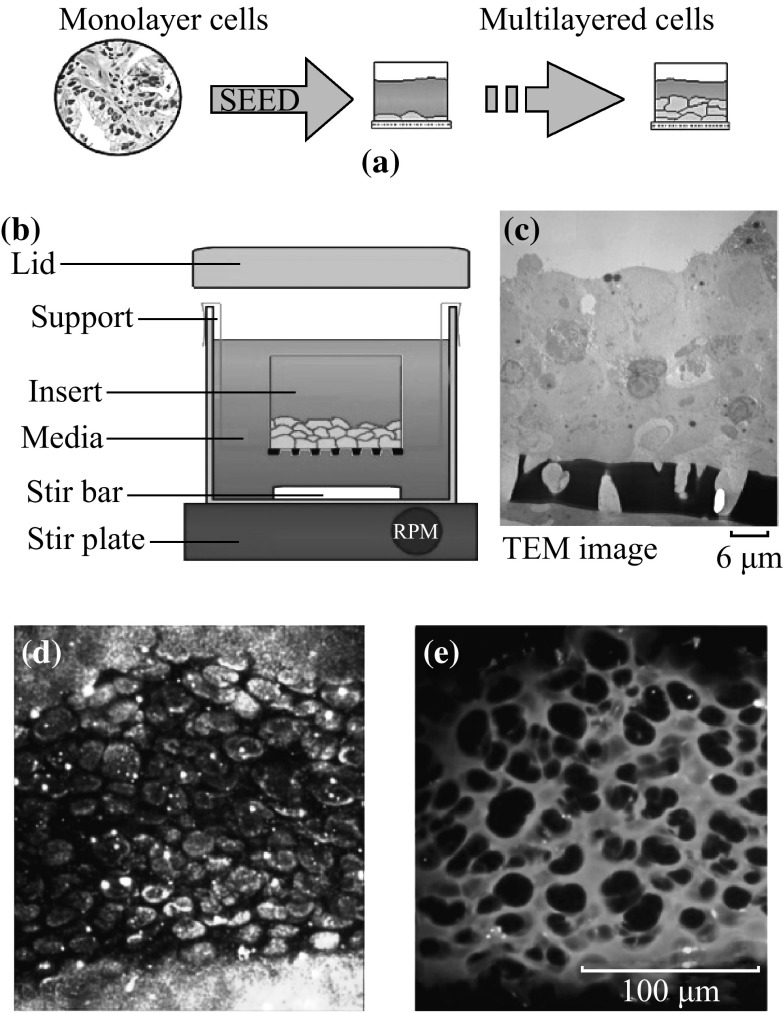
Growth of MCL structures. **a** General principles of MCL growth involve seeding monolayer cells onto a semi-permeable membrane for growth. **b** The basic MCL growth setup showing the insert suspended in stirred media. **c** TEM image of the MCL tissue. **d**, **e** Unstained and stained images of MCL tissue cross sections, respectively

The growth of the MCLs began with the growth of monolayer cells in a 5 % CO_2_ environment at 37 °C. After reaching confluence, these cells were trypsinized, centrifuged, suspended in media, and counted. Approximately 150,000–200,000 cells were seeded onto a microporous membrane insert (Millicell, Bedford, MA) (see Fig. 3a). After allowing the cells to attach for two to four hours, the inserts were washed with PBS to remove unattached cells and then suspended in stirred media to grow (see Fig. 3b). With pore sizes of 3 µm, the inserts allowed for the passage of stirred media through the base of the insert as seen in Fig. 3c. A TEM image of a tissue cross section is shown in Fig. 3c.

Two breast cancer cell lines were used in this study: MCF-7 and MDA-MB-231. Cells were grown on the MLC insert in Dulbecco′s Modified Eagle′s Medium (LifeTechnologies Inc. Burlington, ON) with 10 % Fetal Bovine Serum (Sigma-Aldrich, Oakville, ON). Figure 3d shows an image of an unstained tissue cross section of MCF-7 cells. The ECM and cytoplasm of the cells within the tissue were stained with eosin for visualization (Fig. 3e). The darker areas in the image are the nuclei of cells. The nucleus will not be stained with eosin. The thickness of the tissue was controlled by the growth period. MLC incubation with NPs was conducted by hanging the MLCs in multiwall plates followed by filling the top of the inserts with the GNP and media mixture. A supply of fresh media was placed below the MLC to allow for GNPs that had penetrated the entire MLC structure to diffuse through. MLCs were examined and their thicknesses were measured. The thickness of an MLC was found by taking the average thickness over at least 10 slices.

### Qualitative Analysis

To qualitatively measure the distribution of the GNPs as well as to provide a measure of MLC growth characteristics, MLC inserts were frozen in optimum cutting temperature (OCT) compound for sectioning. The frozen MLCs were then sectioned (Cryostat CM1900; Leica, Wetzlar, Germany) into 10–20-µm-thick sections and placed onto slides for imaging. Tissue sections were stained with eosin to show the presence of ECM (Autostainer XL; Leica, Wetzlar, Germany). Stained tissue sections were imaged using the CytoViva HSI dark-field microscope. By examining the images acquired by HSI, a qualitative examination of the layer-by-layer penetration was deduced.

## Results and Discussion

### Size Dependence of Gold Nanoparticle Uptake at Monolayer Level

**Fig. 4:**
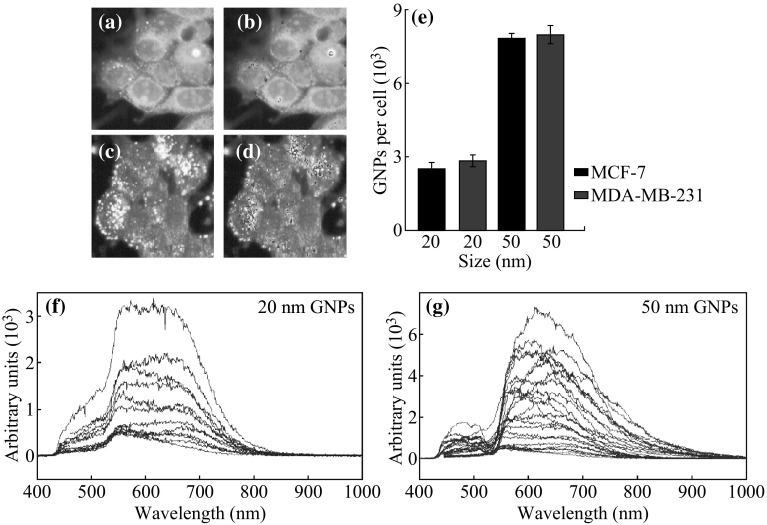
Monolayer uptake of GNPs. **a** The hyperspectral image of cells internalized with 20 nm sized GNPs. *Bright dots* represent GNP clusters localized within cells. **b** GNP clusters within the cells were mapped using one of the reflectance spectra of GNPs. **c** The hyperspectral image of cells internalized with 50 nm sized GNPs. *Bright dots* represent GNP clusters localized within cells. **d** GNP clusters within cells were mapped using the reflectance spectrum of GNPs. **e** The GNP uptake per cell for 20 and 50 nm GNPs across both cell lines. **f**, **g** The reflectance spectra for the 20 and 50 nm GNPs in the monolayer cell samples are shown in (**a**, **c)**

Of particular interest to NP research is the size dependence of NP uptake, which has been confirmed by a number of independent groups [4, 35, 36]. It was found that the uptake of GNPs across a variety of cell lines was highest for 50 nm GNPs, and that smaller (<40 nm) as well as larger (>80 nm) GNPs had almost a two-fold decrease in uptake per cell [4]. We used 20 and 50 nm GNPs for our monolayer and multilayer study. At the monolayer level, our quantitative and qualitative results showed that 20 nm NPs have a lower uptake compared to 50 nm NPs as illustrated in Fig. 4. This is in agreement with the previous studies. This size dependence is explained by the energy dynamics surrounding RME with 50-nm spheres showing a significantly higher uptake than smaller sizes [37, 38]. As particles interact with the surface of the cell, receptors are recruited and bound to the GNP in an exothermic process. This binding of receptors to the surface of the particle provides the cell with the energy required to begin the invagination process that will surround the particle and prepare it for entry into the cell. Smaller particles provide less surface area for receptors to bind, and as a result offer less total energy for the invagination process lowering their uptake. The reflectance spectra from GNPs localized within cells did not show any clear difference for cells incubated with 20 and 50 nm GNPs (see Fig. 4f, g). This could be due to the fact that NPs are in vesicles together following internalization. Hence, it is hard to differentiate reflectance spectra from individual NPs.

### Size Dependence of Gold Nanoparticle Transport at Multilayer Level Using MLC Model

**Fig. 5:**
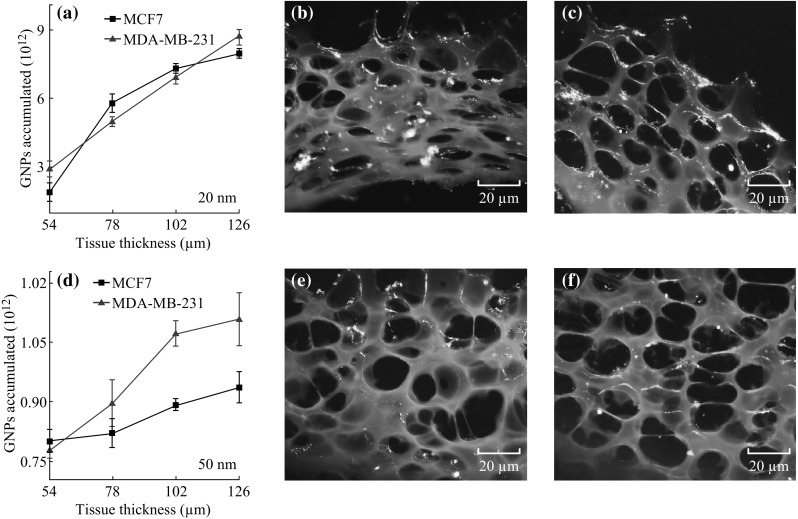
Penetration of GNPs of different sizes in tissue-like MLC structures. **a** The quantitative determination of the penetration of GNPs in MCLs for the 20 size GNPs in MDA-MB-231 and MCF-7 cells. **b**, **c** Dark-field images showing penetration of 20 nm GNPs through MDA-MB-231 and MCF-7 tissues, respectively. **d** The quantitative determination of the penetration of GNPs in MCLs for the 50 size GNPs in MDA-MB-231 and MCF-7 cells. **e**, **f** Dark-field images showing penetration of 50 nm GNPs through MDA-MB-231 and MCF-7 tissues, respectively

**Fig. 6:**
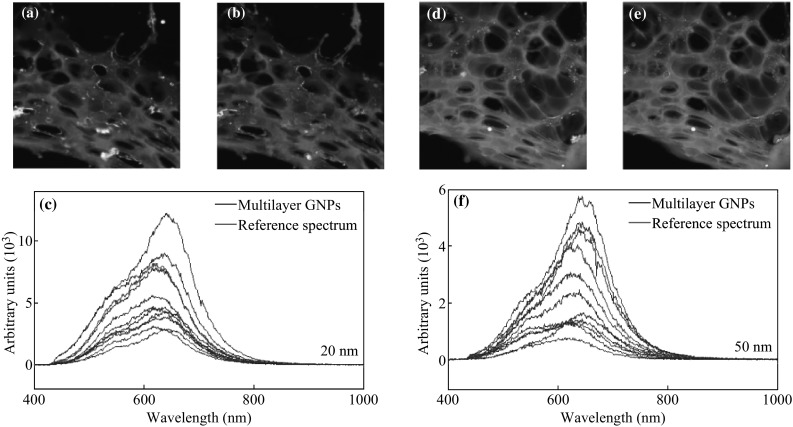
Spectral mapping of size-dependent NP penetration through tissue. **a**, **b** Unmapped and mapped images of 20 nm GNPs penetrated through tissue, respectively. **c** The reflectance spectra obtained for the 20 GNPs localized in the tissue. **d**, **e** Unmapped and mapped images of 50 nm GNPs penetrated through tissue, respectively. **f** The reflectance spectra obtained for the 50 GNPs localized in the tissue

As discussed in the previous section, 50 nm GNPs are preferentially taken up by cells as opposed to smaller GNPs at monolayer level. However, it was unknown as to whether or not this effect would carry over into the tissue-like multilayer model due to the presence of ECM. To test this, 50-nm GNPs were incubated at the same concentration and for the same time period, in MLCs as the 20 nm GNPs. The results of these experiments are shown in Fig. 5. Transport of smaller NPs through MCF-7 and MDA-MB-231 is shown quantitatively and qualitatively in Fig. 5a–c. Smaller NPs seem to have a higher penetration throughout the tissue. However, transport of larger NPs through MCF-7 and MDA-MB-231 is lower as shown in Fig. 5d–f. These results suggest that the optimal particle size dependence of GNP uptake is reversed in the multilayer model in comparison to the monolayer model. Figure 5 shows that the overall penetration of larger GNPs is lower than in smaller GNPs. These results further suggest that the ECM plays a larger role in the transport of GNPs through tissue compared to the individual cells themselves. Penetration of the GNPs was mapped for each NP size using CytoViva HSI dark-field microscope as illustrated in Fig. 6. The qualitative images show that the penetration of smaller GNPs is better than in larger GNPs. This seems to suggest that the diffusive nature of ECM transport is far more pronounced with the 50 nm GNPs. This also highlights the importance of the ECM-limiting effect in the much less structured MDA-MB-231 tissue as opposed to the more ordered and thus more prohibitive MCF-7 tissue. This result is corroborated by several studies that have proposed the effects of collagen content and ECM structure as the major factors in the prohibition of macromolecule penetration through tumor tissue [13, 19–21]. The reflectance spectra from GNPs localized in the ECM and cells were plotted (Fig. 6c, f).

**Fig. 7:**
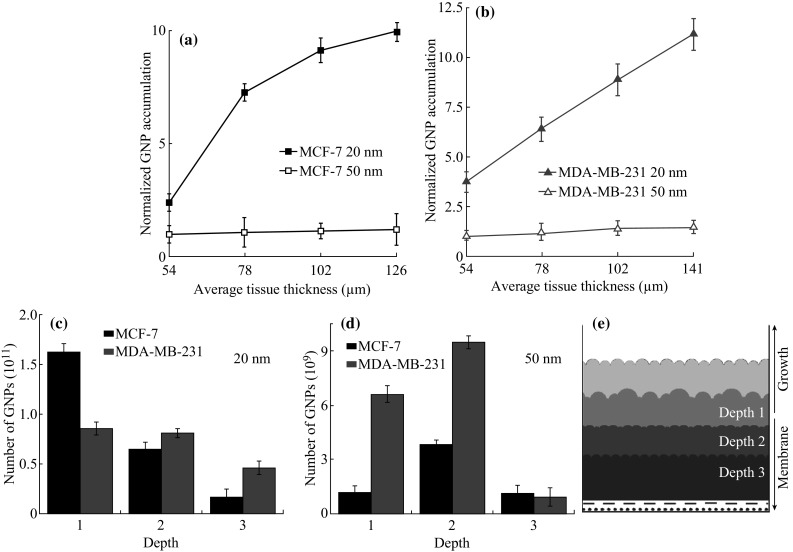
Comparison of NP penetration as a function of size of the NPs. **a**, **b** GNP penetration as a function of size and tissue thickness in MCF-7 and MDA-MB-231 cells, respectively. **c**, **d** Penetration of GNPs as a function of tissue depth for 20 and 50 nm GNPs, respectively. **e** Schematic outlining the various ‘depths’ in MLC tissue structures

Examination of the rate of change in accumulation as a function of tissue thickness (Fig. 7a, b) provides a qualitative look at the penetration of GNPs in the MCL tissue. The transport of NPs as a function of depth was also analyzed as illustrated in Fig. 7c, d. The depth profile used is shown in Fig. 7e. For the 20 nm GNPs, it appears that the particles were able to penetrate and accumulate in the deeper tissue. For 50 nm GNPs, the overall accumulation and penetration is lower for both cell lines. Furthermore, there is an increase in penetration of up to approximately 100 µm of thickness. However, there is then a very rapid falloff in penetrative ability across both cell lines for the 50 nm GNPs. This could indicate that 100 µm is the maximum penetrative distance of 50 nm GNPs in tissue-like materials developed. The slow diffusion of larger NPs through tumor tissue could be due to the presence of ECM, as discussed below.

In epithelial cells, the ECM combines the use of a highly organized collagen network, hyaluronic acid, and a number of proteins to prohibit the passage of macromolecules through tissue. Transport through the ECM is largely mediated via diffusion, and thus, the ECM is less permeable to particles of larger sizes as given by the Stokes–Einstein equation:

$$D = \frac{{k_{\text{b}} T}}{{6\pi \mu R_{\text{h}} }}$$

In the equation above, *D* is the diffusion coefficient in m s^−1^ (often cm s^−1^), *k*_b_ as the Boltzmann consent, *T* as the temperature in K, *µ* as the viscosity in kg (s m)^−1^, and *R*_h_ as the hydrodynamic radius of the passing particle in m. This relation assumes spherical particles of a homogenous material with a low Reynolds number and no interaction between the particles. As such, it is not a perfect representation of the present situation but it provides a baseline model to predict the overall trends in particle penetration as a function of particle size.

Several studies have noted that the limiting effects of the ECM are tied to collagen content and the structural organization of the ECM itself [7, 8]. Although convection through the interstitial matrix is an important part of interstitial fluid and particle movement, it is limited by the hypertension found in the interstitium [11, 22, 39]. Many anticancer agents such as liposomes and gene vectors are large molecules ranging in size from 90 to 300 nm. Nanoparticle agents being developed also vary greatly in size ranging from 2 to 200 nm. Thus, diffusion through the interstitial matrix of the tumor presents a major barrier to the delivery of therapeutic agents and drugs.

In addition, cancer cells have been shown to disrupt and change the composition and makeup of the ECM. Invasive species in particular have been shown to break down the collagen that helps provide the ECM with its ability to limit the flow of foreign particles through tissue [25, 26]. The two cell lines used in this study are thus separated by the makeup and composition of their respective ECM. The measurements taken will establish what effect the ECM has on the transport of GNPs in tissue. According to Fig. 7c–d, NP penetration is better in deeper tissues in MDA-MB-231 in comparison to MCF-7. This could be due to the difference in ECM in these tumor tissues. One other factor to consider when discussing the penetration dynamics in solid tumors is the tortuosity of the path taken by particles through tumor tissue. Particles traversing or penetrating tissue are presented with cellular obstacles that force the particles on tortuous paths through the interstitial space [19, 31]. The tortuosity is not only difficult to measure but also will vary greatly between cell lines and also within a single tumor [19].

## Conclusions

**Fig. 8:**
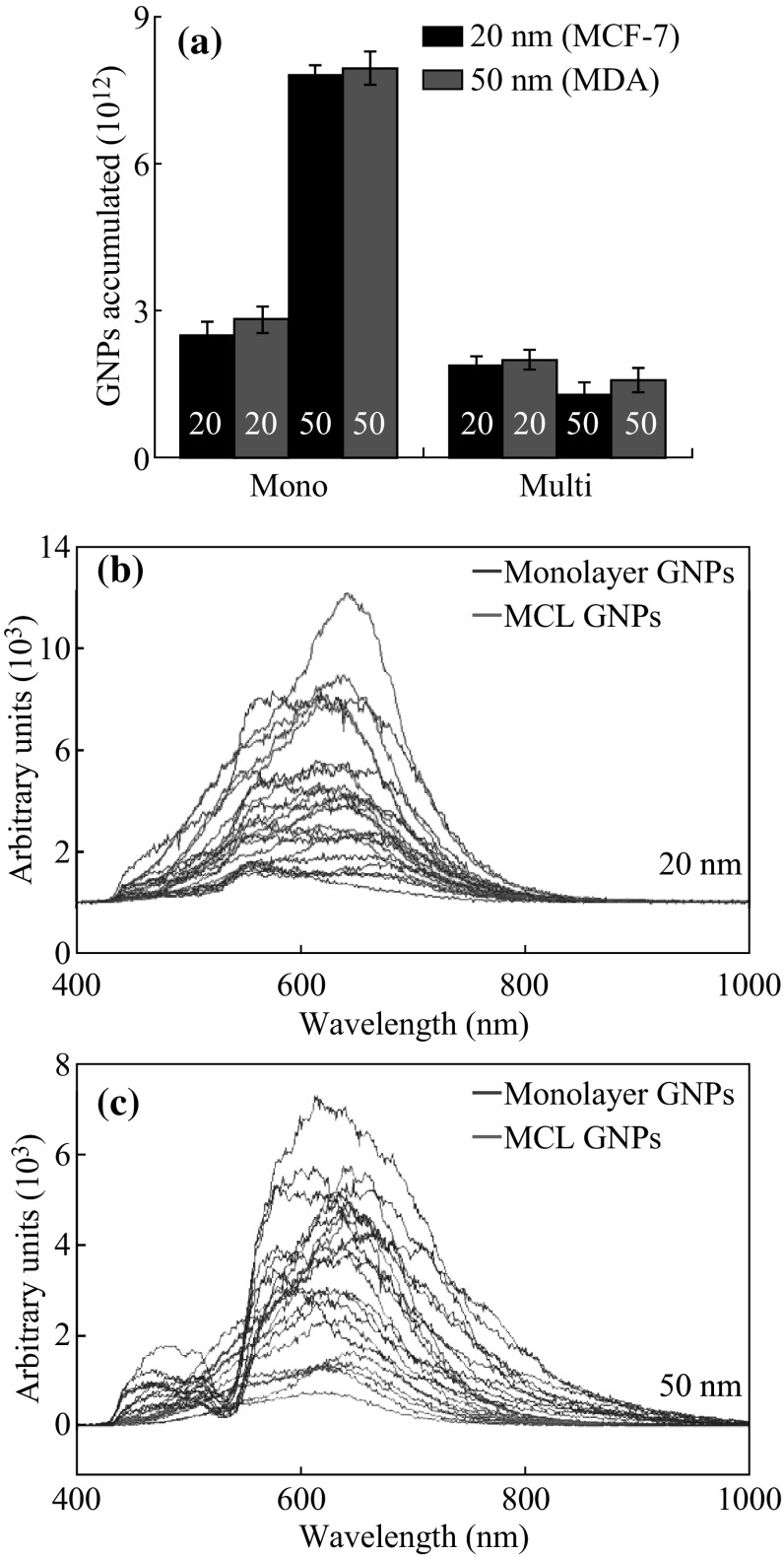
Comparison of size-dependent NP uptake at monolayer and multilayer level. **a** The comparison of uptake per cell between monolayer and multilayer cultures across all cell lines for both sizes of GNPs. **b** The scaled reflectance spectra from the 20 nm GNPs for both the monolayer (*blue*) and multilayer (*red*) samples. **c** The scaled reflectance spectra from the 50 nm GNPs for the monolayer (*blue*) and multilayer (*red*) samples. (Color figure online)

The size dependence of GNP uptake at the monolayer level appears to be reversed in the multilayer structures. Figure 8a summarizes the results and compares the uptake per cell for the monolayer and multilayer study. Figure 8b, c shows the reflectance spectra for both sizes of the GNPs across both cell lines. Both sizes of GNPs show broader peaks in the monolayer cultures. This could be the result of the clustering of GNPs that occurs upon entry into the cells where they are packaged into lysosomes. This result also suggests that GNPs may be traversing the ECM one at a time or at least in lower quantities than they are produced when GNPs are clustered into lysosomes. The results suggest that GNP or NP systems that aim to improve particle passage through the ECM might perform better in the in vivo environment. So far, the design of GNP systems has largely rested on achieving better GNP–cell interactions and improving the outcomes of RME. This design philosophy ignores the effects of the ECM which has been shown here to be potentially as important a factor in the transport of GNPs in solid tumors as the cells themselves. Based on nanoparticle penetration in tissue-like materials, smaller NPs would be better since it allows deeper tumor penetration. In the future, ligand-bound GNP systems can be tested with the MLC model to determine which ligands actively improve the overall accumulation of GNPs at depth in solid tumors.

## Electronic supplementary material

Below is the link to the electronic supplementary material.

(TIF 296 kb)
