# Supplementary material for: Size-Dependent Gold Nanoparticle Interaction at Nano–Micro Interface Using Both Monolayer and Multilayer (Tissue-Like) Cell Models
Source: Nanomicro Lett. 2015 Sep 15;8(1):44–53. doi: 10.1007/s40820-015-0060-6 (PMC6223926; doi:10.1007/s40820-015-0060-6)
Supplement: Supplementary file 1 — (TIF 296 kb) [file 40820_2015_60_MOESM1_ESM.docx]

Supplementary Information for

**Size Dependent Gold Nanoparticle Interaction at Nano-Micro Interface Using both Monolayer and Multilayer (Tissue-Like) Cell Models**

Darren Yohan^1^, Charmainne Cruje^1^, Xiaofeng Lu^2^, Devika B. Chithrani^1, 2,^ ⃰

^1^Department of Physics, Ryerson University, 350 Victoria Street, Toronto, ON, Canada, M5B 2K3

^2^ Keenan Research Centre, Li Ka Shing Knowledge Institute, St. Michael's Hospital, Toronto, ON, Canada

⃰ Corresponding author. E-mail: [devika.chithrani@ryerson.ca](mailto:devika.chithrani@ryerson.ca)

Tel.: 416 979 5000 ext. 4115; Fax: 416 979 5000

**S1 Characterization of Gold Nanoparticles (GNPs)**

We used Dynamic Light Scattering (DLS), Transmission Electron Microscopy (TEM), and UV visible spectroscopy to characterize the size distribution of GNPs used for our experiments.

1. TEM images of 20 and 50 nm diameter GNPs

Fig. S1 TEM images of 20 (left) and 50 nm (right) diameter GNPs.

The scale bar represents 100 nm

1. Table summarizing the TEM, DLS, and UV data for 20 and 50 nm GNPs

|  | UV abruption Wavelength (nm) | Diameter (nm)  TEM | Diameter (nm)  DLS |
| --- | --- | --- | --- |
| 20 nm | 520 | 19.5 ± 3.3 | 24.2 ± 0.6 |
| 50 nm | 525 | 52.4 ± 5.2 | 52.6 ± 0.8 |
